# ROBot-assisted physical training of older patients during acUte hospitaliSaTion—study protocol for a randomised controlled trial (ROBUST)

**DOI:** 10.1186/s13063-024-08044-6

**Published:** 2024-04-04

**Authors:** Ann Sophia Bertelsen, Tahir Masud, Charlotte Suetta, Lisbeth Rosenbek Minet, Stig Andersen, Jørgen T. Lauridsen, Jesper Ryg

**Affiliations:** 1 Geriatric Research Unit, Department of Clinical Research, University of Southern Denmark, Odense, Denmark; 2 Department of Geriatric Medicine, Odense University Hospital, Odense, Denmark; 3 Open Patient Data Explorative Network, OPEN, Odense University Hospital, Region of Southern Denmark, Odense, Denmark; 4 Department of Geriatric Medicine, Nottingham University Hospital NHS Trust, Nottingham, UK; 5 Department of Geriatric and Palliative Medicine, Copenhagen University Hospital, Bispebjerg and Frederiksberg, Copenhagen, Denmark; 6 Department of Clinical Medicine, Faculty of Health, University of Copenhagen, Copenhagen, Denmark; 7 Department of Geriatric Medicine, Aalborg University Hospital, Aalborg, Denmark; 8 Department of Economics, University of Southern Denmark, Odense, Denmark

**Keywords:** Geriatrics, Functional status, Hospitalisation, Inactivity, RCT, ADL, Barthel index, Sarcopenia, Chair stand test

## Abstract

**Background:**

During hospitalisation, older patients spend most of their time passive in bed, which increases the risk of functional decline and negative adverse outcomes. Our aim is to examine the impact of robot-assisted physical training on functional status in older geriatric patients during acute hospitalisation.

**Methods:**

This is a single-centre investigator-blinded placebo-controlled randomised controlled trial including geriatric patients aged ≥ 65 years, able to ambulate before hospitalisation, and with expected length of stay ≥ 2 days. In addition to standard physiotherapy treatment, the intervention group receive active robot-assisted resistance training and the control group passive robot-assisted sham training. Exclusion criteria are as follows: ambulation without assistance at the time of inclusion, known severe dementia, delirium, patients who have received less than three training sessions at discharge, terminal illness, recent major surgery/lower extremity fracture, conditions contradicting the use of training robot, lower extremity metastases, deemed unsuitable for robot-assisted training by a healthcare professional, or weight > 165 kg. The primary outcome is functional status assessed by change in Barthel Index-100 and 30-s chair stand test between inclusion and day of discharge. Secondary outcomes include functional status at 1- and 3-month follow-up, quality of life, depression, concern about falling, falls, cognition, qualitative interviews, need of homecare, discharge destination, readmissions, healthcare costs, sarcopenia, muscle quantity (bioimpedance), and mortality.

Clinical meaningful change of the Barthel Index is 5 points. A recent study in geriatric patients reported a 6.9-point change following exercise. With a significance level of 5%, 80% power, and a drop-out rate of 20%, 244 participants per group (*n* = 488) are needed to detect the same mean difference. With a significance level of 5%, 80% power, and a drop-out rate of 20%, 74 participants per group (*n* = 148) are needed to detect a minimum clinical change of 2.6 repetitions for 30-s chair stand test. Recruitment started in January 2023 and is expected to continue for 19 months including follow-up.

**Discussion:**

If our study shows that in-hospital robot-assisted training prevents functional decline in older patients, this may have a major impact on the individual patient due to increased wellbeing and a higher level of independency. In addition, society will benefit due to potential decrease in the need of municipality-delivered homecare following discharge.

**Trial registration:**

ClinicalTrials.gov NCT05782855. Registration date: March 24, 2023.

## Administrative information

**Table:** 

Title {1}	ROBot-assisted physical training of older patients during acUte hospitaliSaTion—study protocol for a randomised controlled trial (ROBUST)
Trial registration {2a and 2b}	ClinicalTrials.gov: NCT05782855
Protocol version {3}	Initial released at ClinicalTrials.gov: December 27, 2022 Last update at ClinicalTrials.gov: December 5, 2023
Funding {4}	This study is supported by grants from The Independent Research Fund Denmark; The Health Foundation, Denmark; the Svend Andersen Foundation, Denmark; the Gangsted Foundation, Denmark; Odense University Hospital Fund for Free Research, Denmark; The Region of Southern Denmark; Department of Geriatric Medicine, Svendborg Hospital, Denmark; Department of Geriatric Medicine, Odense, Denmark
Author details {5a}	1: Geriatric Research Unit, Department of Clinical Research, University of Southern Denmark 2: Department of Geriatric Medicine, Odense University Hospital, Denmark 3: OPEN, Open Patient data Explorative Network, Odense University Hospital, Region of Southern Denmark, Odense C, Denmark 4: Department of Geriatric Medicine, Nottingham University Hospital NHS Trust, Nottingham, UK 5: Department of Geriatric and Palliative Medicine, Copenhagen University Hospital, Bispebjerg and Frederiksberg, Copenhagen, Denmark 6: Department of Clinical Medicine, Faculty of Health, University of Copenhagen, Copenhagen, Denmark 7: Department of Geriatric Medicine, Aalborg University Hospital, Denmark 8: Department of Economics, University of Southern Denmark
Name and contact information for the trial sponsor {5b}	Jesper Ryg, MD, Professor, PhD Department of Geriatric Medicine Odense University Hospital J.B. Winsloews Vej 4 5000 Odense C Denmark E-mail: Jesper.Ryg@rsyd.dk
Role of sponsor {5c}	Not applicable

### Introduction

#### Background and rationale {6a}

Hospitalisation is associated with a high risk of loss of independence especially in older patients living with frailty [1]. Hospital-associated disability leads to the inability to ambulate, prolonged hospitalisation, higher health care expenditures, and increased requirement for institutionalisation after discharge [1]. Functional decline is the leading complication of hospitalisation in older people where at least 34% experience a loss of independence in at least one basic activity of daily living as an unintended consequence of their hospital stay [1, 2]. A major reason is that older people spend most of their time passive in bed while hospitalised [3, 4]. Whereas physical inactivity poses a threat to muscle tissue and functional capacity to all people, older adults lose lean tissue most rapidly. Loss of muscle mass in bedridden patients happens fast [5] and more than half of all older people do not recover their pre-admission functional level 1 year after discharge with high rates of care home admissions and increased mortality [4, 6, 7].

Studies have shown that in-hospital exercise and early physical rehabilitation are beneficial for older people in terms of improved physical functioning, shorter hospital stay, and reduced care home admissions [8, 9]. Furthermore, a reduction in length of stay can decrease in-patient hospital costs and increase hospital bed availability, increasing the overall cost-efficiency of hospitals [10]. Despite this, early mobilisation and training of the least active older people is often overlooked as an intervention during hospitalisation [1].

The field of robot technology in rehabilitation is expanding with an increase in new devices and technologies emerging each year [11]. Robots have the potential to increase the quantity of therapy received by an individual. One such example is the newly developed Danish training robot ROBERT® [12]. This robot is capable of helping patients to perform exercises while the patient is lying in bed, which makes it possible to train even bedridden people effectively.

We previously performed and published a pilot test and feasibility study using ROBERT® version 1.0. A version performing only passive mobilisation [13]. 74% of the approached eligible older patients agreed to participate in the pilot and feasibility study and expressed that they would recommend the training robot. The study showed that the use of robot technology in the passive mobilisation of older patients was feasible and well-accepted by patients, relatives, and staff. However, in order to be able to train patients actively, a customised training device was warranted. Therefore, the training robot was updated and further improved following our pilot and feasibility study. In this way, the newest version is now able to perform both passive and active movements enabling patients to do resistance training.

To ensure patient and public involvement, we asked our research reference group of older patients, relatives, municipalities, and relevant stakeholders about their input on relevant outcomes in a clinical trial of older patients during hospitalisation. The group highlighted the importance of avoiding functional decline during hospitalisation by maintaining physical independence and psychological wellbeing. However, in many countries including Denmark, intensive rehabilitation training has been moved away from hospitals during the period of acute illness to the subsequent convalescence period in the municipalities following hospital discharge. Therefore, we regularly miss the opportunity to prevent and intervene against hospital-associated functional decline.

To start an early physical rehabilitation programme, knowledge of which patient population benefit from the programme is required. Furthermore, it is important to know if there will be adverse events during early physical rehabilitation programmes in terms of falls or other injuries and what the adherence rate of the patients will be during the treatment sessions [9]. Previous studies found that early rehabilitation programmes improve both patient (e.g. physical functioning) and hospital outcomes (e.g. reducing costs) for acute ill geriatric patients. However, an important issue not yet addressed in the current literature is the feasibility of in-hospital early robot exercise programmes for acute geriatric patients along with randomised controlled trial (RCT) studies using sham intervention to proper investigation of effect.

### Objectives {7}

The main purpose of this study is to examine the impact of robot-assisted physical training on functional status during acute hospitalisation in older geriatric patients.

### Trial design {8}

The ROBUST study is an investigator-blinded placebo-controlled RCT in older patients acutely admitted to a geriatric department with 3-month follow-up. All participants (*n* = 488) will receive standard individual physiotherapy and care during hospitalisation. Participants will be randomised in parallel groups to either robot-assisted active resistance training (active group) or robot-assisted passive sham training (control group) in a 1:1 ratio with blocking without stratification. The RCT has a superiority framework.

## Methods: participants, interventions, and outcomes

### Study setting {9}

The study is a single-centre study, which takes place at the Department of Geriatric Medicine, Odense University Hospital, Svendborg, Denmark. The department is a 32-bed unit with ~ 2000 yearly acutely admitted older patients with multimorbidity and polypharmacy. Median length of stay is 6 days. The department receives patients from the hospital emergency department, geriatric outpatient clinic, and transfers from other hospital departments.

### Eligibility criteria {10}

Older people admitted to the Department of Geriatric Medicine at Odense University Hospital, Svendborg, Denmark will be eligible for study participation.

Inclusion criteria:

-  ≥ 65 years of age
- Able to ambulate before hospitalisation (with/without assistance)
- Able to communicate with the research team
- Expected length of stay ≥ 2 days
- Residing on Funen

Exclusion criteria at the time of baseline enrolment:

- Able to ambulate without assistance at the time of inclusion
- Known severe dementia
- Delirium defined as positive Confusion and Assessment Method score [14]
- Patients who have received less than 3 training sessions at discharge
- Terminal illness
- Recent major surgery or lower extremity bone fracture in the past 3 months
- Conditions contradicting the use of ROBERT® (unstable vertebral, pelvic, or lower extremity fractures; high intracranial pressure; pressure ulcers or risk of developing pressure ulcers due to fragile skin; patients with medical instability)
- Metastases at femur or hip
- Deemed not suitable for mobilisation sessions with the robot by the healthcare professional
- Weight > 165 kg

### Who will take informed consent? {26a}

A clinically experienced healthcare professional will be responsible for the recruitment of patients. Written and verbal informed consent will be obtained. Any patient who declines to participate in the study will be respected. The patient need not to give any reason for decline and this will not have any impact on their further treatment during hospitalisation.

### Additional consent provisions for collection and use of participant data and biological specimens {26b}

A signed consent form will indicate whether participants give their permission for the use of their data in the study. Information on standard clinical blood samples is collected from medical health records. No extra biological specimens are collected in the trial.

## Interventions

### Explanation for the choice of comparators {6b}

Exercise and early rehabilitation are beneficial for older people in terms of improved physical functioning, shorter hospital stay, and reduced care home admissions [8, 9]. Thus, to evaluate whether robot-assisted physical resistance training can be a good strategy to mitigate the negative outcomes of inactivity during hospitalisation for this population, passive training movements by the robot are considered as a sham control group for comparing the effect of the active resistance training intervention group. Sham controls provide the highest quality and potentially most generalisable clinical trial data. This setup is particularly useful when studying devices controlling for the ancillary effects of a procedure, optimising the ability of the investigator to evaluate for a placebo or procedural effect in an unbiased fashion [15].

### Intervention description {11a}

**Fig. 1:**
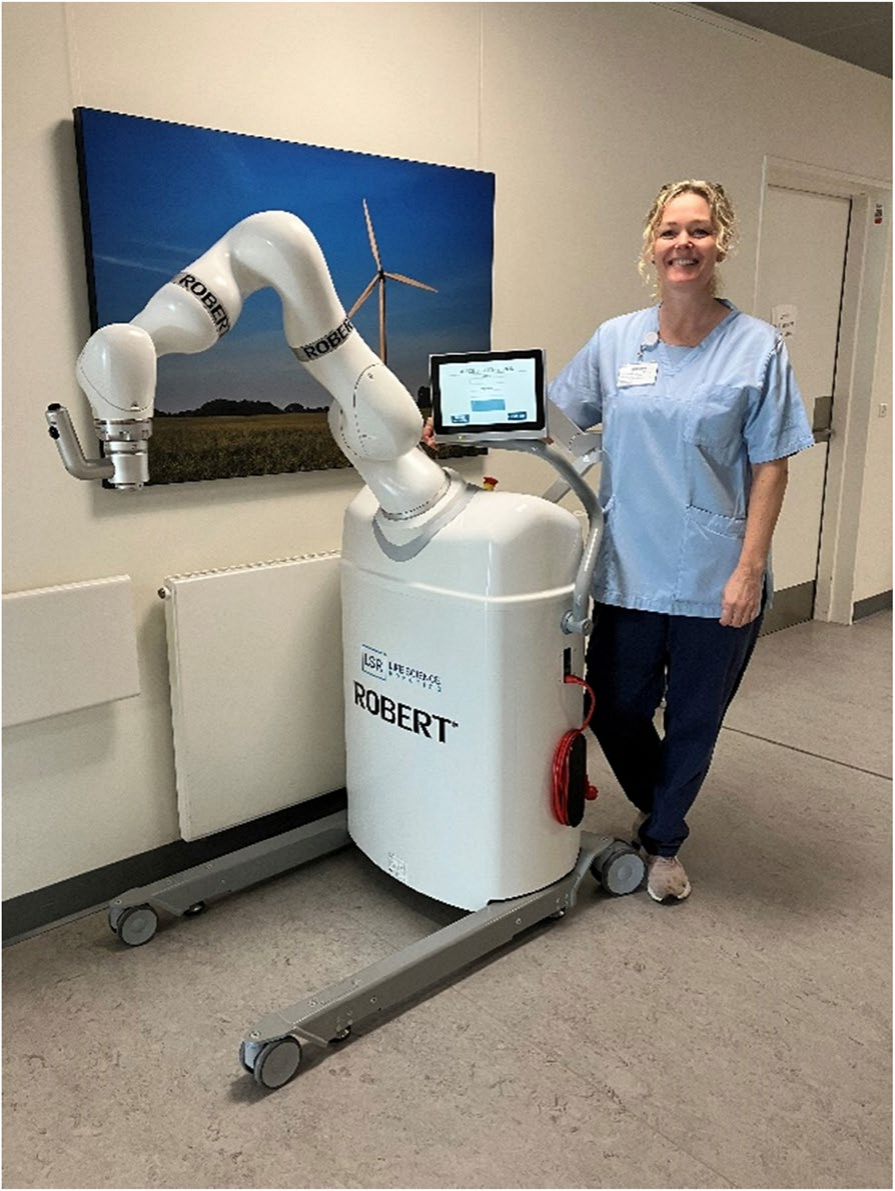
ROBERT®

**Fig. 2:**
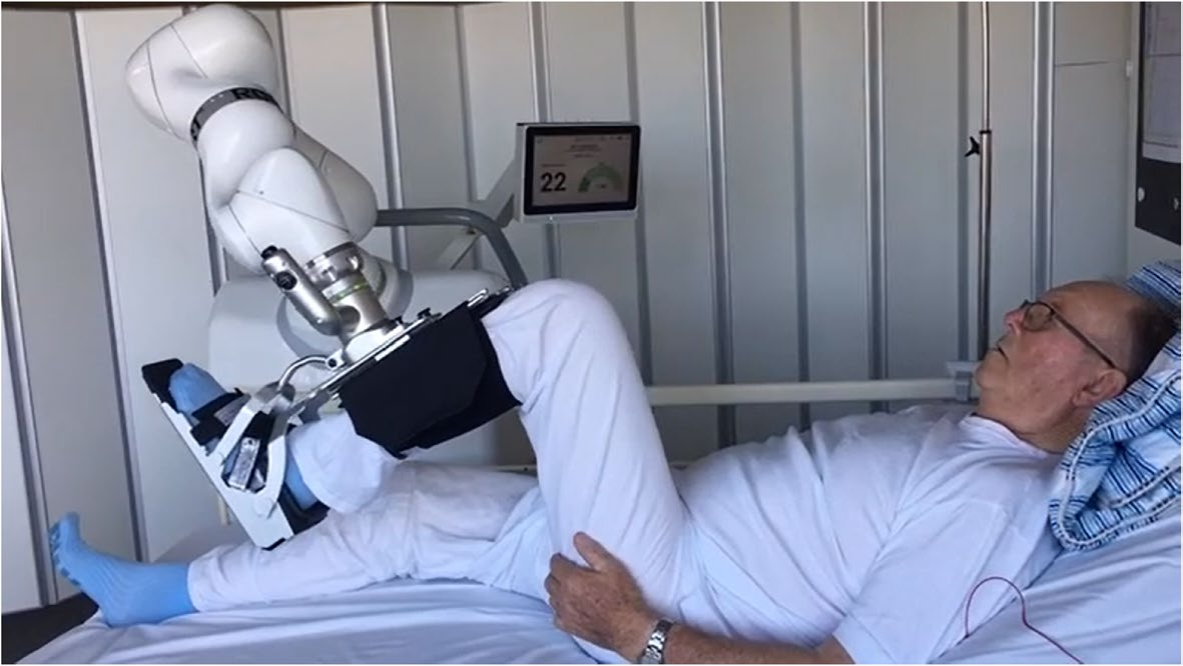
ROBERT® can hold the patient’s leg and perform extension of hip and knee

The robot-assisted training is performed using an innovative training robot (ROBERT®) (Fig. 1). The robot is able to hold the patient’s leg and perform extension movements of the hip and knee (Fig. 2). The movement can be performed either active or passive. In the active training mode, the patient must use their muscular power to stretch the leg while the robot provides low/moderate resistance. In the passive mode, the robot moves the leg independently without the patient using their own muscle power. Exercise is performed on both legs separately. The robot-assisted training is defined as a minimum of three sessions during the hospital stay. Following each training session, all participants are offered nutritional drinkable supplements each containing a minimum of 18 to 26 g of protein per serving (125–250 ml) [16, 17].

#### Intervention group

The intervention group will receive active robot-assisted resistance training twice a day until the day of discharge in addition to standard individualised care and physiotherapy. A resistance training session consists of three sets of active extensions of the hip and knee under verbal motivation in order to perform as many repetitions as possible. Training intensity is 65–100% of maximum capacity with a break of 60 s between each set. To ensure progression, the level of resistance is assessed at each session. The Borg scale is used for ratings of perceived exertion following every training set [18].

#### Control group

Participants in the control group will receive passive robot-assisted sham training by the robot twice a day until the day of discharge in addition to standard individualised care and physiotherapy. A sham training session consists of three sets of eight repetitions performed by the training robot, which passively moves the patient’s leg in extension of the hip and knee with a break of 60 s between each set.

### Criteria for discontinuing or modifying allocated interventions {11b}

Participants will discontinue allocated intervention if there is a deconditioning in their health state or if they develop contradictions for robot training during their in-hospital stay. Also, the intervention will terminate if a participant is referred to another hospital.

Any patient requesting to terminate their study participation can be withdrawn from the study regardless of the stage they have reached in the study process. Patients do not have to provide the reason of their study withdrawal.

### Strategies to improve adherence to interventions {11c}

Appointed research staff are present in the department every day to carry out screening, inclusion, and robot training with the patients. The research staff will encourage patients to continue daily training (both sham and resistance) to improve adherence and furthermore collect data on the amount of robot training. This will ensure daily motivation for continued participation. Reasons for discontinuing participation will be collected.

### Relevant concomitant care permitted or prohibited during the trial {11d}

The robot training will not require alteration to usual care pathways for participants during hospitalisation or following discharge (including the use of any medication or physical activity).

### Provisions for post-trial care {30}

All patients who participate in this study are covered by Danish Patient Compensation if they suffer harm from trial participation [19].

### Outcomes {12}

Outcomes in the current study are selected based on inputs from patient public involvement. During study preparation patients, relatives, and stakeholders including representatives from the municipality’s health care setting participated in a workshop with the aim of designating research focus areas. The group rated functional independence and quality of life as the most important outcomes of interest.

#### Baseline data

Demographic data will be collected at baseline to describe the study population and assess potential subgroup differences. This includes the following data: age, gender, civil- and living status, body mass index (BMI), use of medications, C-reactive protein (CRP) blood sample, historic Barthel Index (14 days before hospital admission), and reason for hospital admission (ICD-10 diagnose [20]).

#### Primary outcome

The primary outcome of interest is functional status defined as activities of daily living (ADL) measured by Barthel and sit-to-stand performance measured by 30-s chair stand test from baseline prior to randomisation and to the day of discharge. ADL characterise the capability of a person to do everyday routine activities. The current study uses the Barthel Index-100, which is a recognised and simple scoring instrument used to evaluate basic ADL functions, the level of physical performance, and the intensity of needed care [21]. The Barthel Index is often used as a gold standard comparator in studies addressing ADL [22]. It is a sum score across ten domains of ADL and the total score ranges from 0 (completely dependent) to 100 (completely independent) [23]. Sit-to-stand performance is assessed as the number of times a person is able to rise and sit from a standardised chair within 30 s [24].

### Secondary outcomes

Several relevant secondary outcomes are assessed.

#### Functional status

Functional status as secondary outcome is assessed by Barthel Index-100 and sit-to-stand performance at baseline before randomisation and at 1- and 3-month follow-up.

#### Discharge destination

Discharge destination will be collected (own home, temporary rehabilitation units, care homes).

#### Need of care at home

Individual level data from the municipalities will be used to assess amount of home care provided by the municipality during the period of 3 months before admission to 3 months after discharge. Need of care at home is divided into categories of practical help, personnel care, nursing, and training. Amount of care will be presented in hours of provided assistance.

#### Muscle quantity

Muscle quantity is assessed using bioelectrical impedance by InBodyS10® [25] at baseline before randomisation, at discharge, and at 1- and 3-month follow-up. Bioelectrical impedance analysis (BIA) is a method for estimating body composition, in particular body fat and muscle mass.

#### Sarcopenia

Sarcopenia is assessed based on the 2019 European guidelines [26] at baseline before randomisation, at discharge, and at 1- and 3-month follow-up.

#### Clinical frailty scale

The 9-point Clinical Frailty Scale (CFS) with pictograms is used at baseline before randomisation, at discharge, and at 1- and 3-month follow-up [27] to examine the impact of the exercise intervention on frailty and to determine the impact of baseline frailty on the effectiveness of the intervention [28].

#### Quality of life

Quality of life is assessed using the questionnaire “Quality of life EuroQol-5 dimension (EQ-5D)” (patient-reported outcome (PRO)) at baseline before randomisation, at discharge, and at 1- and 3-month follow-up.

The EQ-5D comprises five dimensions (mobility, self-care, usual activities, pain/discomfort, and anxiety/depression). The answers given to EQ-5D generate 243 unique health states or can be converted into an EQ-5D index, a utility score anchored at 0 for death and 1 for perfect health. The EQ-5D questionnaire also includes a visual analogue scale (VAS), by which respondents can report their perceived health status with a grade ranging from 0 (the worst possible health status) to 100 (the best possible health status) [29, 30].

#### Concern about falling

Concern about falling is assessed using the 16-item Short Falls Efficacy Scale International (Short FES-I) questionnaire (patient-reported outcome (PRO)) [31, 32] including information on the actual number of falls. Assessments are performed at baseline before randomisation, at discharge, and at 1- and 3-month follow-up.

#### Cognition function

Cognitive function is assessed by the Mini-Mental State Examination (MMSE) at baseline before randomisation, at discharge, and at 1- and 3-month follow-up. The MMSE is a set of 11 questions used to assess potential cognitive impairment (problems with thinking, communication, understanding, and memory). The maximum score for the MMSE is 30. A score of 25 or higher is classed as normal. If the score is ≤ 24, the result is usually considered to be abnormal, indicating possible cognitive impairment.

#### Mood

Mood status is assessed by the 15-item Geriatric Depression Scale (GDS) at baseline before randomisation, at discharge, and at 1- and 3-month follow-up. The short form GDS consists of 15 questions. Of the 15 items, 10 indicate the presence of depressive symptoms when answered positively, while the rest indicate depressive symptoms when answered negatively. Scores of 0–4 are considered normal, depending on age, education, and complaints; 5–8 indicate mild depression; 9–11 moderate depression; and 12–15 indicate severe depression [33].

#### Hospitalisation

Length of hospital stay (LOS) will be defined as the number of days in the geriatric department. Readmission is defined as any unplanned hospital contact with a duration of 12 + h, occurring between 4 h and 30 days following discharge from the geriatric department [34]. Data will be collected using a medical records review.

#### Mortality

Mortality is assessed during hospital stay and at 1- and 3-month follow-up.

#### Patient perspective

Participant observation and qualitative semi-structured interviews will be performed with 10–12 patients during the trial period to explore patient perspectives and experiences (patient-reported experience (PRE)). The qualitative interviews will provide additional information to the results from the quantitative parts of the study. By using this mixed methods approach the research results will be strengthened by the complementary findings. The analysis of the qualitative data will be completed in line with manifest content analysis by Graneheim and Lundman [35].

#### Health care cost evaluation

A researcher in health care economics will perform a health care cost evaluation addressing running costs using the training robot, discharge destination, hospital readmissions, need of care at home, and visits to the general practitioner after 3 months.

### Participant timeline {13}

Patients will be enrolled and allocated within a maximum of 48 h from the time of admission to the geriatric department. Each day patients are listed in order according to most recently admitted and screened according to this list. When finding an eligible patient, the research assistant immediately asks the patient about participation and starts the baseline tests if the patient has agreed and signed the consent form. After baseline test completion, the screening continues. Daily screening ends when logistic capacity does not allow further daily participation. After baseline tests, patients will be allocated randomly to either active robot training or passive robot training. At discharge, the dates for 1- and 3-month follow-up are arranged with the patient. The schedule of enrolment, interventions, and assessments are shown schematically in Table 1.

**Table 1:** SPIRIT—schedule of enrolment, interventions, and assessments

	**Enrolment**	**Allocation**	**Close-out**		
**Timepoint**	** − t**_**1**_	**Admission**	**Discharge**	**1-month follow-up**	**3-month follow-up**
**Enrolment:**					
** Eligibility screening**	X				
** Informed consent**	X				
** Demographic data**		X			
**Age, gender, civil- and living status, body mass index (BMI)**		X			
**Allocation**		X			
**Intervention received from admission to discharge:**					
** Active resistance robot training**		X	X		
** Passive sham robot training**		X	X		
**Assessments:**					
** Current Barthel-Index**		X	X	X	X
** Historical Barthel-Index**		X			
** 30-s chair stand test**		X	X	X	X
**Quality of life EuroQol-5 dimension (EQ-5D)**		X	X	X	X
**Mini-Mental State Examination (MMSE)**		X	X	X	X
**Short Falls Efficacy Scale International (Short FES-I)**		X	X	X	X
**Number of falls**		X	X	X	X
**Geriatric Depression Scale (GDS)**		X	X	X	X
**Muscle quantity (bioimpedance by InBodyS10**®**)**		X	X	X	X
**Sarcopenia**		X	X	X	X
**Need of homecare**		X	X	X	X
**Clinical Frailty Scale (CFS)**		X	X	X	X
**Length of hospital stay (LOS)**			X		
**Discharge destination**			X		
**Hospital readmission**				X	
**Mortality**			X	X	X
**C-reactive protein (CRP) Blood sample**		X			
**Reason for admission (ICD-10 diagnose)**			X		
**Use of medications**		X	X		

### Sample size {14}

The minimum clinical important change for the 30-s chair stand test is 2.6 repetitions [36] and the mean reference value for people in the relevant age groups is 13 according to a recent Danish study [37]. With a significance level of 5%, 80% power, and an expected drop-out rate of 20%, 74 participants in each group (148 in total) are needed to detect an inter-group difference of 2.6 repetitions. The magnitude of clinical meaningful change of the Barthel Index is 5 points and a recent study in geriatric patients reported a change of 6.9 points during exercise [6]. The mean (SD) Barthel Index score of patients at the geriatric department OUH is 59.5 (24.3). To achieve 80% power for demonstrating the same mean difference, this study would require 244 participants per group (488 in total) with a significance level of 5% and an expected drop-out rate of 20%.

Thus, a total of 488 participants is required for our primary outcome.

### Recruitment {15}

A total of 74% of the approached eligible older patients agreed to participate in our previously performed pilot and feasibility study and expressed that they would recommend the training robot [13]. Thus, our study group already has experience using a training robot in geriatric patients. Our department is a 32-bed unit with ~ 2000 patients admitted each year. If we assume only 25% of the patients will be eligible for the trial and accept participation a realistic inclusion period to obtain 488 participants is 16 months.

## Assignment of interventions: allocation

### Sequence generation {16a}

Patients will be allocated randomly to either the control or intervention group with a 1:1 allocation and blocking using computer-generated randomisation set up by a statistician from Open Patient data Explorative Network (OPEN), Odense University Hospital, Region of Southern Denmark [38]. No stratification is applied. Blocking information is only available to the daily project manager. No information regarding blocking is given to those recruiting participants, assigning the intervention, or collecting outcome data.

### Concealment mechanism {16b}

The randomisation sequence will be automatically generated by the software program REDCap (Research Electronic Data Capture) (version: REDCap 12.0.33—© 2022 Vanderbilt University) hosted by OPEN at Odense University Hospital, Region of Southern Denmark and shared with the project manager. Allocation concealment will be guaranteed, as REDCap will withhold the randomisation code until the patient is recruited into the trial. Additionally, all staff responsible for collecting outcomes during the intervention will be blinded to the randomisation process.

### Implementation {16c}

Clinically experienced healthcare professionals will screen, enrol participants, and perform outcome tests blinded to allocation. The daily project manager will initiate the automated randomisation and allocate participants to the control or intervention group. The trainers who will monitor the robot-assisted physical training of patients can see the allocation in REDCap, thus they can program the robot to do either active or passive training. The trainers will not do any outcome tests or other tasks in the study.

## Assignment of interventions: blinding

### Who will be blinded {17a}

The study is an investigator-blinded placebo-controlled RCT. All trial participants will receive either sham or active robot-assisted training. The health care workers responsible for assessing all outcomes in the hospital are blinded to allocation. Hospital staff not linked to the study are blinded to allocation. Also, all data will be analysed blinded to allocation.

### Procedure for unblinding if needed {17b}

If patients experience severe adverse events they will be unblinded to ensure safety measures.

## Data collection and management

### Plans for assessment and collection of outcomes {18a}

The time points of patient recruitment, intervention, data collection, processing, and follow-up operations are described in Table 1 and study instruments in Sect. 12 about the assessed outcomes. The collected data will be entered into the electronic data system of REDcap.

All testing personnel were trained to collect outcome measures according to relevant guidelines. Furthermore, each tester practised data collecting before initiation.

### Plans to promote participant retention and complete follow-up {18b}

For participant retention and complete follow-up, the research team will make motivational conversations with the patients during their admission and at follow-up home visits to reinforce the importance of the study programme and evaluations. Furthermore, a telephone reminder will be given to each participant the day before follow-up to ensure data collection. Patients who have completed less than three training sessions during their entire admission will only have primary outcome tests performed at discharge and no follow-up.

### Data management {19}

In accordance with Danish laws on data protection, study data will be collected and managed using REDCap electronic data capture tools hosted at OPEN, Open Patient data Explorative Network, Odense University Hospital, Region of Southern Denmark (43, 44). REDCap is a secure, web-based software platform designed to support data capture for research studies, providing (1) an intuitive interface for validated data capture; (2) audit trails for tracking data manipulation and export procedures; (3) automated export procedures for seamless data downloads to common statistical packages; and (4) procedures for data integration and interoperability with external sources [39].

No paperwork will be stored. The data management plan is available from the corresponding author upon request.

### Confidentiality {27}

All sensitive data from participants will be archived in REDCap and in a secure SharePoint webpage approved by the Danish Data Protection Agency (21/3398). Each participant will receive a numerical code and personal data will be obliterated in the data analysis. All research documents will be saved for 5 years following publication and will hereafter be deleted.

### Plans for collection, laboratory evaluation and storage of biological specimens for genetic or molecular analysis in this trial/future use {33}

There will be no biological specimens collected in the trial (see 26b).

## Statistical methods

### Statistical methods for primary and secondary outcomes {20a}

The characteristics of the two groups will be compared by descriptive statistics. Variables will be described as means (+ / −) with standard deviations (SD) when normally distributed or medians with quartiles (IQR) when not. Variables measured serially during follow-up will be analysed by chi-square or Fisher’s test.

The randomised groups will be compared with respect to the primary and secondary endpoints in an intention-to-treat analysis. A predefined per-protocol analysis will also be performed. Linear mixed-effects regression models will be used to assess differences from baseline to follow-up. Otherwise, survival will be assessed by Kaplan–Meier analysis and hospital readmissions with regression models. The level of statistical significance will be 0.05.

### Interim analyses {21b}

No interim analyses are pre-planned.

### Methods for additional analyses (e.g. subgroup analyses) {20b}

The following subgroup analyses are pre-planned:

- Difference between groups in relation to the amount of completed training sessions (> 75%)
- Time to rehabilitation in the municipality after discharge
- Difference compared to baseline functional level (Barthel, CFS, home care before admission)
- Difference compared to performance in 30-s chair stand test

The subgroup analyses aim at determining whether the overall effect varies across different patient characteristics/ across subgroups of clinical importance.

### Methods in analysis to handle protocol non-adherence and any statistical methods to handle missing data {20c}

Regarding non-adherence, a subgroup analysis of participants training < 75% is already pre-planned (20b). No imputation will be made in case of missing data, which means the fully adjusted models are conducted as complete case analyses.

### Plans to give access to the full protocol, participant-level data and statistical code {31c}

Due to Danish legislation, public access to information on participant level is prohibited. Information on statistical codes may be provided by the corresponding author upon reasonable request.

## Oversight and monitoring

### Composition of the coordinating centre and trial steering committee {5d}

This is a single-centre study. The coordinating centre of this study is the Department of Geriatric Medicine, Odense University Hospital directed by the primary investigator and main supervisor Professor Dr. Jesper Ryg. The daily project manager is PhD student Ann Sophia Bertelsen, MSPH, who will be responsible for coordinating the intervention programme, including recruiting and enrolling patients, organising and conducting assessments, data management, and data analysis. The main supervisor and PhD student meet weekly/monthly according to need. A joint meeting with the total supervisor group is held every 6 months. The daily project manager meets with the research team (assessors and/or trainers) on a weekly basis.

### Composition of the data monitoring committee, its role and reporting structure {21a}

Ethics committee and regulatory authorities do not require DMC for the current study since this is a low-risk intervention exploring a CE-approved medical device. Life Science Robotics ApS is ISO-13485 certified and ROBERT® is approved as a Class IIa medical device in compliance with the medical device directive (93/42/EEC) [12]. The use is within the scope of what the device is developed for.

### Adverse event reporting and harms {22}

Some patients may experience short-term, slight redness of the skin where the robot accessory has been located. If the robot accessory has been tightened too much or for a long time, the patient’s toes may begin to feel numb. This stops shortly after the accessory is removed.

Adverse events (AEs) are defined as any untoward or unfavourable medical occurrences associated with the subject’s participation during the research. All AEs will be recorded in detail. Once any adverse reaction occurs during robot training, the training will be stopped immediately. Serious AEs will be reported to the Danish Medicines Agency, the study principal investigator, and the manufacturer immediately, and appropriate measures will be initiated instantly. The Danish Medicines Agency will determine whether the AE is likely to have been associated with the experimental robot training and whether it is necessary to break the blinding codes.

### Frequency and plans for auditing trial conduct {23}

No formal auditing is planned for this trial. However, the trial is audited in an ongoing manner to ensure patient safety. Every 6 months, there will be a report on the recruitment rate, data quality, protocol deviations, and adverse events. In case of serious adverse events, these will be sent for audit within 2 weeks. The events are audited independently from the trial investigators by The Regional Scientific Ethical Committee of Southern Denmark.

### Plans for communicating important protocol amendments to relevant parties (e.g. trial participants, ethical committees) {25}

Any changes or amendments in protocol will be reviewed by the whole study group and be approved by the local ethics committee. Any modifications will be reported, and the trial protocol will be updated.

### Dissemination plans {31a}

The results will be disseminated to the scientific community and relevant groups via publications in scientific journals, presentations at conferences, and reporting of the results in databases (ClinicalTrials.gov) and on social media.

## Discussion

If our study finds that the use of a training robot improves the functional status of older people following hospitalisation it may have a major impact on the individual patient due to increased wellbeing and a higher level of independency. In addition, society will benefit due to a potential decrease in the need of care in the municipality following discharge from the hospital. It is expected that the majority of older people admitted to medical wards benefit from the training robot and thus experience positive gains from this training. In this way, the study is expected to help change clinical practice for optimal in-hospital rehabilitation of older persons.

### Limitations

Limitations of the study can be divided into (1) “inclusion limitation” and (2) “design limitations”. (1) The assessed group of patients in the current study are characterised by high age, frailty, multimorbidity, and polypharmacy upon their acute illness. It might therefore be expected that only a few patients will accept participation. However, in our pilot study, 74% of the approached patients, accepted to participate. In the current study, only 25% acceptance is required to have enough power to complete the study. (2) In order to minimise bias, we have designed an investigator-blinded placebo-controlled study where both patient groups perform training with the robot. Whether we can ensure blinding to allocation group for each participant during their study participation is uncertain. However, blinding for outcome performers and data analysis will be kept.

### Trial status

Protocol version: December 5, 2023.

Ethical approval: June 29, 2021 (Project-ID: S-20210029)

Recruitment initiation: January 5, 2023

Anticipated recruitment finalisation: May 31, 2024

## Data Availability

JR and ASB will have full access to the final trial dataset.

## Abbreviations

ADL: Activities of daily life
BIA: Bioelectrical impedance analysis
BMI: Body mass index
CFS: Clinical Frailty Scale
CRP: C-reactive protein
EQ-5D: Quality of life EuroQol-5 dimension
FES-I: Falls Efficacy Scale International
GDS: Geriatric Depression Scale
ICD-10: International Classification of Diseases 10th Revision
LOS: Length of hospital stay
MMSE: Mini-Mental State Examination
PRE: Patient-reported experience
PRO: Patient-reported outcome
RCT: Randomised controlled trial
REDCap: Research Electronic Data Capture
ROBUST: ROBot-assisted physical training of older patients during acUte hospitalisation
VAS: Visual analogue scale

## References

[1] Surkan MJ, Gibson W (2018). Interventions to mobilize elderly patients and reduce length of hospital stay. Can J Cardiol.

[2] Covinsky KE, Pierluissi E, Johnston CB (2011). Hospitalization-associated disability: "She was probably able to ambulate, but I'm not sure". JAMA.

[3] Brown CJ, Redden DT, Flood KL, Allman RM (2009). The underrecognized epidemic of low mobility during hospitalization of older adults. J Am Geriatr Soc.

[4] Greysen SR (2016). Activating Hospitalized Older Patients to Confront the Epidemic of Low Mobility. JAMA Intern Med.

[5] Reidy PT, Lindsay CC, McKenzie AI, Fry CS, Supiano MA, Marcus RL (2018). Aging-related effects of bed rest followed by eccentric exercise rehabilitation on skeletal muscle macrophages and insulin sensitivity. Exp Gerontol.

[6] Martínez-Velilla N, Casas-Herrero A, Zambom-Ferraresi F, Sáez de Asteasu ML, Lucia A, Galbete A, et al. Effect of Exercise Intervention on Functional Decline in Very Elderly Patients During Acute Hospitalization: A Randomized Clinical Trial. JAMA Internal Medicine. 2019;179(1):28–36. 10.1001/jamainternmed.2018.4869 PMC6583412 30419096

[7] Boyd CM, Landefeld CS, Counsell SR, Palmer RM, Fortinsky RH, Kresevic D (2008). Recovery of activities of daily living in older adults after hospitalization for acute medical illness. J Am Geriatr Soc.

[8] Lim SER, Ibrahim K, Sayer AA, Roberts HC (2018). Assessment of Physical Activity of Hospitalised Older Adults: A Systematic Review. J Nutr Health Aging.

[9] Kosse NM, Dutmer AL, Dasenbrock L, Bauer JM, Lamoth CJ (2013). Effectiveness and feasibility of early physical rehabilitation programs for geriatric hospitalized patients: a systematic review. BMC Geriatr.

[10] Heldmann P, Werner C, Belala N, Bauer JM, Hauer K (2019). Early inpatient rehabilitation for acutely hospitalized older patients: a systematic review of outcome measures. BMC Geriatr.

[11] Yakub F, Md Khudzari AZ, Mori Y (2014). Recent trends for practical rehabilitation robotics, current challenges and the future. Int J Rehabil Res.

[12] Lifescience-robotics. Lifescience-robotics 2022 [Available from: https://www.lifescience-robotics.com/. Assessed 19 Feb 2024.

[13] Bertelsen AS, Storm A, Minet L, Ryg J (2020). Use of robot technology in passive mobilization of acute hospitalized geriatric medicine patients: a pilot test and feasibility study. Pilot and feasibility studies.

[14] Inouye SK, van Dyck CH, Alessi CA, Balkin S, Siegal AP, Horwitz RI (1990). Clarifying confusion: the confusion assessment method. A new method for detection of delirium. Ann Intern Med.

[15] Sutherland ER (2007). Sham procedure versus usual care as the control in clinical trials of devices: which is better?. Proc Am Thorac Soc.

[16] Nowson C, O'Connell S (2015). Protein Requirements and Recommendations for Older People: A Review. Nutrients.

[17] Coelho-Junior HJ, Marzetti E, Picca A, Cesari M, Uchida MC, Calvani R. Protein Intake and Frailty: A Matter of Quantity, Quality, and Timing. Nutrients. 2020;12(10). 10.3390/nu12102915 PMC7598653 32977714

[18] Borg GA (1982). Psychophysical bases of perceived exertion. Med Sci Sports Exerc.

[19] Compensation DP. Danish Patient Compensation [Available from: https://eng.patienterstatningen.dk/. Assessed 19 Feb 2024.

[20] World_Health_Organization. International Statistical Classification of Diseases and Related Health Problems 10th Revision 2019 [Available from: https://icd.who.int/browse10/2019/en. Assessed 19 Feb 2024.

[21] Katz for the Association of Rheumatology Health Professionals Outcomes Measures Task Force PP. Measures of adult general functional status: The Barthel Index, Katz Index of Activities of Daily Living, Health Assessment Questionnaire (HAQ), MACTAR Patient Preference Disability Questionnaire, and Modified Health Assessment Questionnaire (MHAQ). Arthritis Care & Research. 2003;49(S5):S15-S27.

[22] Quinn TJ, Langhorne P, Stott DJ (2011). Barthel index for stroke trials: development, properties, and application. Stroke.

[23] Shah S, Vanclay F, Cooper B (1989). Improving the sensitivity of the Barthel Index for stroke rehabilitation. J Clin Epidemiol.

[24] Jones CJ, Rikli RE, Beam WC (1999). A 30-s chair-stand test as a measure of lower body strength in community-residing older adults. Res Q Exerc Sport.

[25] INBODY_DANMARK. INBODYS10 [Available from: https://inbodydanmark.dk/produkter/inbodys10/. Assessed 19 Feb 2024.

[26] Cruz-Jentoft AJ, Bahat G, Bauer J, Boirie Y, Bruyère O, Cederholm T (2019). Sarcopenia: revised European consensus on definition and diagnosis. Age Ageing.

[27] Fournaise A, Nissen SK, Lauridsen JT, Ryg J, Nickel CH, Gudex C (2021). Translation of the updated clinical frailty scale 2.0 into Danish and implications for cross-sectoral reliability. BMC geriatrics.

[28] Pérez-Zepeda MU, Martínez-Velilla N, Kehler DS, Izquierdo M, Rockwood K, Theou O. The impact of an exercise intervention on frailty levels in hospitalised older adults: secondary analysis of a randomised controlled trial. Age and ageing. 2022;51(2). 10.1093/ageing/afac028 35180287

[29] EuroQol--a new facility for the measurement of health-related quality of life. Health Policy. 1990;16(3):199–208. 10.1016/0168-8510(90)90421-9 10109801

[30] Wittrup-Jensen KU, Lauridsen J, Gudex C, Pedersen KM (2009). Generation of a Danish TTO value set for EQ-5D health states. Scandinavian journal of public health.

[31] Yardley L, Beyer N, Hauer K, Kempen G, Piot-Ziegler C, Todd C (2005). Development and initial validation of the Falls Efficacy Scale-International (FES-I). Age Ageing.

[32] Kempen GI, Yardley L, van Haastregt JC, Zijlstra GA, Beyer N, Hauer K (2008). The Short FES-I: a shortened version of the falls efficacy scale-international to assess fear of falling. Age Ageing.

[33] Yesavage JA, Brink TL, Rose TL, Lum O, Huang V, Adey M (1982). Development and validation of a geriatric depression screening scale: a preliminary report. J Psychiatr Res.

[34] Thomsen K, Fournaise A, Matzen LE, Andersen-Ranberg K, Ryg J (2021). Does geriatric follow-up visits reduce hospital readmission among older patients discharged to temporary care at a skilled nursing facility: a before-and-after cohort study. BMJ Open.

[35] Graneheim UH, Lundman B (2004). Qualitative content analysis in nursing research: concepts, procedures and measures to achieve trustworthiness. Nurse Educ Today.

[36] Wright AA, Cook CE, Baxter GD, Dockerty JD, Abbott JH (2011). A comparison of 3 methodological approaches to defining major clinically important improvement of 4 performance measures in patients with hip osteoarthritis. J Orthop Sports Phys Ther.

[37] Suetta C, Haddock B, Alcazar J, Noerst T, Hansen OM, Ludvig H (2019). The Copenhagen Sarcopenia Study: lean mass, strength, power, and physical function in a Danish cohort aged 20–93 years. J Cachexia Sarcopenia Muscle.

[38] Open_Patient_Data_Explorative_Network. OPEN, Open Patient data Explorative Network: OPEN, Open Patient data Explorative Network, Odense University Hospital, Region of Southern Denmark; [Available from: https://open.rsyd.dk/. Assessed 19 Feb 2024.

[39] REDCap(ResearchElectronicDataCapture). REDCap (Research Electronic Data Capture) October 2023 [Available from: https://projectredcap.org/software/. Assessed 19 Feb 2024.

